# 3-Dimensional shear wave elastography of breast lesions: Added value of color patterns with emphasis on crater sign of coronal plane

**DOI:** 10.1097/MD.0000000000004877

**Published:** 2016-09-30

**Authors:** Ya-ling Chen, Cai Chang, Wei Zeng, Fen Wang, Jia-jian Chen, Ning Qu

**Affiliations:** a Department of Ultrasound, Fudan University Shanghai Cancer Center; b Department of Oncology, Shanghai Medical College, Fudan University; c Department of Breast Surgery; d Department of Head and Neck Surgery, Fudan University Shanghai Cancer Center, Shanghai, China.

**Keywords:** breast, elastography, shear wave, 3-dimensional, ultrasound

## Abstract

Color patterns of 3-dimensional (3D) shear wave elastography (SWE) is a promising method in differentiating tumoral nodules recently. This study was to evaluate the diagnostic accuracy of color patterns of 3D SWE in breast lesions, with special emphasis on coronal planes.

A total of 198 consecutive women with 198 breast lesions (125 malignant and 73 benign) were included, who underwent conventional ultrasound (US), 3D B-mode, and 3D SWE before surgical excision. SWE color patterns of Views A (transverse), T (sagittal), and C (coronal) were determined. Sensitivity, specificity, and the area under the receiver operating characteristic curve (AUC) were calculated.

Distribution of SWE color patterns was significantly different between malignant and benign lesions (*P* = 0.001). In malignant lesions, “Stiff Rim” was significantly more frequent in View C (crater sign, 60.8%) than in View A (51.2%, *P* = 0.013) and View T (54.1%, *P* = 0.035). AUC for combination of “Crater Sign” and conventional US was significantly higher than View A (0.929 vs 0.902, *P* = 0.004) and View T (0.929 vs 0.907, *P* = 0.009), and specificity significantly increased (90.4% vs 78.1%, *P* = 0.013) without significant change in sensitivity (85.6% vs 88.0%, *P* = 0.664) as compared with conventional US.

In conclusion, combination of conventional US with 3D SWE color patterns significantly increased diagnostic accuracy, with “Crater Sign” in coronal plane of the highest value.

## 1 Introduction

Breast ultrasonic elastography has become a routine tool in addition to conventional B-mode ultrasound (US) during the last few years. Although strain elastography with freehand compression was demonstrated useful for differential diagnosis of breast lesions, with sensitivity of 79% to 98% and specificity of 72% to 88%,^[1,2]^ the technique itself had several obvious limitations such as operator-dependent, less reproducible, and lack of quantitative information about elasticity modulus.^[3]^ Shear wave elastography (SWE), as a brand new method of elastography, induces shear waves that transversely propagate in the tissue, using an acoustic radiation force created by a focused US beam. SWE could provide both qualitative and quantitative elastic information in real time,^[3]^ which was proved to be of highly intra- and interobserver reproducibility in breast lesions.^[4]^

Previous studies have demonstrated the diagnostic performance of SWE in breast lesions, among which the prospective multicenter, multinational study—BE1—was of the largest sample.^[5]^ The important conclusion was that adding SWE features to conventional B-mode analysis improved specificity of breast mass assessment (78.5% vs 61.1%, *P* < 0.001) without loss of sensitivity. Other studies showed similar results of improved diagnostic performance with increased specificity,^[6–8]^ could increase positive predictive values for nonmass lesions in breast,^[9]^ and thus could reduce unnecessary biopsies of low-suspicion Breast Imaging Reporting and Data System (BI-RADS) category 4A masses. Besides, several studies have indicated the correlation between SWE quantitative features and histologic prognostic factors—breast cancers with higher mean stiffness values at SWE had poorer prognostic features.^[10,11]^

The diagnostic values of SWE features mentioned above were for 2-dimensional (2D) SWE, while there were only 2 published studies in 3-dimensional (3D) SWE of breast lesions so far, which demonstrated that diagnostic accuracy of 3D SWE was no better than 2D SWE.^[12,13]^ However, both the 2 studies focused on comparison of quantitative features of SWE, without emphasis on qualitative features, especially comparison of color patterns among transverse, sagittal, and coronal planes. As 1 important qualitative feature, color patterns of SWE first proposed by Tozaki and Fukuma^[14]^ was proved to be useful in differential diagnosis of breast lesions, and diagnostic value of “Stiff Rim” sign, as 1 type of the color patterns of SWE indicating malignancy, was emphasized by Zhou et al.^[15]^ Therefore, the purpose of our study was to determine whether 3 views reconstructed by 3D SWE could provide more information about color patterns and thus improve diagnostic performance, with emphasis on that of coronal planes.

## 2 Materials and methods

### 2.1 Patients

This retrospective study was approved by the Institutional Review Board of Fudan University Shanghai Cancer Center, and verbal informed consent was provided by all participating women at times of examinations. From May 2014 to August 2014, 210 consecutive women with 210 breast masses detected by palpation and/or imaging were enrolled, who underwent examinations of conventional 2D US, 3D B-mode, and 3D SWE before surgical excision. Five patients with history of ipsilateral breast surgery and 1 patient with breast implants were excluded, and so were 6 patients with large masses (over 4 cm) which could not be covered by the maximum range of SWE color overlay and thus may impair judgment of SWE color patterns of breast lesions. Finally, 198 women (mean age, 49.1 ± 11.1 years; age range, 24–84 years) with 198 breast masses constituted the study cohort.

### 2.2 Image acquisition

Conventional US and SWE examinations were performed using the Aixplorer US system (SuperSonic Imagine, Aix-en-Provence, France) by 1 of 3 radiologists with 5 to 20 years of experience in breast imaging. All participating investigators were experienced in performing and/or interpreting over 4000 breast US examinations in the prior 2 years and have practiced SWE on a minimum of 200 cases over the last 6 months. Conventional 2D US and color Doppler was performed with a SL15–4 multifrequency linear-array transducer. We first used the default preset of breast, with center frequency at “GEN” mode, median frequency rate, dynamic range at 70 dB, acoustic power 0.0 dB, and tissue tuner 1480 m/s. When scanning lesions with deep location, we downgraded the center frequency to “PEN” mode, while upgraded to “RES” with superficial location. The color scale was preset at 4 cm/s. For each breast mass, at least 2 orthogonal B-mode images, 1 color Doppler flow image, and 1 pulsed wave Doppler image when necessary were acquired and saved for analysis.

3D B-mode and SWE were performed with a SLV16–5 transducer, which was very lightly applied to avoid compression and kept still during 3D B-mode and SWE data acquisition. The volume scan was automatically performed by using a slow-tilt movement of the sectorial mechanical transducer. Immediately after data acquisition, volume data were reconstructed and displayed in 3 orthogonal planes—transverse (View A), sagittal (View T), and coronal (View C), and then converted to multislice display mode (slice gap, 0.5–0.9 mm; slab thickness, 0–0.25 mm) to have a full view of each slice in 3 planes.^[12,13]^ 3D SWE was carried out with the scale setting at default value—180 kPa.

### 2.3 Conventional US image analysis

Before 3D B-mode and SWE imaging, independent and blinded review of conventional US images of all lesions was performed by 2 principle investigators with 20 years of experience in breast US and classified into appropriate categories according to the American College of Radiology (ACR) BI-RADS to indicate probability of malignancy: BI-RADS Category 3 indicated probably benign; Category 4A, low suspicion for malignancy; Category 4B, moderate suspicion for malignancy; Category 4C, high suspicion for malignancy; and Category 5, highly suggestive of malignancy.^[16,17]^ When the 2 principle investigators did not reach agreement on the classification, another experienced radiologist was invited to discuss and finally reach a consensus.

### 2.4 3D B-mode image analysis and hypothetical effect on BI-RADS assessment

**Figure 1:**
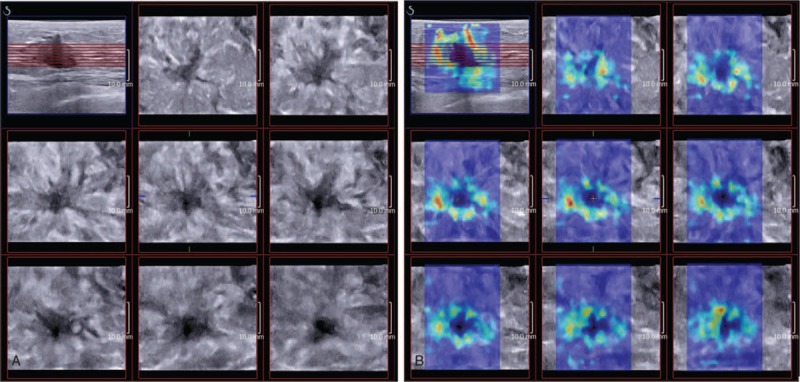
Images showed reconstructed coronal planes of 3D B-mode and 3D shear wave elastography of Grade II invasive ductal carcinoma of a 40-year-old woman. Image (A) showed “Converging Pattern” of coronal plane in multislice mode, defined as hyperechoic bands of fibrous tissue converging toward the hypoechoic mass. Image (B) showed “Crater Sign” in multislice mode, defined as a colored “Stiff Rim” surrounding the mass in coronal plane.

The reconstructed coronal planes were observed carefully slice by slice to identify the presence of the “Converging Pattern” in the surrounding tissue and the margin of the lesion, which was highly suggestive of malignancy and defined as hyperechoic bands of fibrous tissue converging toward the hypoechoic central core of the mass (Fig. 1).^[21]^ To analyze hypothetical effect of 3D B-mode imaging on diagnostic performance of BI-RADS Category assessment, BI-RADS Categories adjacent to cutoff value of diagnosing benign and malignant lesions were regulated according to presence of “Converging Pattern” (upgraded if presence and downgraded if not).

### 2.5 3D SWE image analysis and hypothetical effect on BI-RADS assessment

Each lesion was observed successively in transverse (View A), sagittal (View T), and coronal (View C) planes in multislice mode to determine the SWE color pattern, including mainly 4 patterns proposed by Tozaki and Fukuma as follows: Pattern 1 (no findings), the lesion and surrounding tissue both displaying as homogeneously blue without visual difference in between; Pattern 2 (vertical stripes), light blue or green stripes extending beyond the lesion and continuing vertically in cords on the cutaneous side or the thoracic wall side; Pattern 3 (stiff rim), a localized colored area appeared at the margin of the lesion and created a continuous closed circle; and Pattern 4 (colored lesion), heterogeneously colored areas present in the interior of the lesion.^[14]^ There were other SWE color patterns defined by BE1 investigators: Pattern 5 (horseshoe), a localized colored area at the margin of the lesion which created an open circle; and Pattern 6 (spots above/below), colored areas visible above and/or below the lesion. Yet another pattern described by BE1 was “Void Area Inside the Lesion”, defined as a lack of SWE signal inside the lesion while the rest of the SWE Box filled correctly, which in our study was included into Pattern 3 if closed circle appeared at the margin or into Pattern 5 if colored area created an open circle.^[5]^ In each plane, the slice showing the maximal diameter of the lesion was selected to determine the SWE color patterns of Views A, T, and C, respectively. Independent and blinded review of 3D SWE images of all lesions was performed by 2 principle investigators with 20 years of experience in breast US to determine the SWE color patterns. Another experienced radiologist was invited to discuss and finally reach a consensus when the 2 principle investigators did not reach agreement.

As proposed by Tozaki and Fukuma,^[14]^ Patterns 1 and 2 were assumed to be benign, and Patterns 3 and 4 were assumed to be malignant. BI-RADS Categories adjacent to cutoff value were adjusted according to SWE color pattern (upgraded if SWE pattern was “Stiff Rim” or “Colored Lesions” while downgraded if SWE pattern was “No findings” and “Vertical Stripes”). BI-RADS Categories did not change with other patterns.

### 2.6 Crater sign in coronal plane

The diagnostic performance of “Stiff Rim” Pattern alone was specially analyzed. BI-RADS Categories adjacent to cutoff value were regulated according to the presence of “Stiff Rim” (upgraded if presence and downgraded if not).

As mentioned above, malignant breast lesions frequently showed as “Converging Pattern” in coronal plane, thus making lesions of “Stiff Rim” Pattern in coronal plane (View C) present as a mass surrounded by a colored rim with radiating hyperechoic bands and appear like craters. So in our study, “Stiff Rim” in coronal plane (View C) was renamed as “Crater Sign”.

### 2.7 Diagnostic performance of coronal plane, combining SWE with B-mode

When we combined SWE with B-mode of coronal plane (parallel test), BI-RADS Categories were upgraded with the presence of 1 of “Converging Pattern” and “Crater Sign” or both the 2, while downgraded without the presence of “Converging Pattern” nor “Crater Sign”. The diagnostic performance of adjusted BI-RADS Categories was analyzed.

### 2.8 Histopathologic examination

All the lesions enrolled underwent surgical excision, and histopathologic outcome was used as the Golden Standard. Final diagnosis for each lesion was made by a pathologist with 20 years of experience in breast pathology who was blinded to the US results.

### 2.9 Statistical analysis

Statistical analyses were performed by using SPSS, version 19.0 (SPSS, Chicago, IL). To evaluate diagnostic performance, receiver operating characteristic (ROC) curves was analyzed by using MedCalc for Windows, version 13.1.2.0 (MedCalc Software, Mariakerke, Belgium). Sensitivity, specificity, and area under the ROC curve (AUC) were calculated. The optimal cutoff values were determined by using the Youden index. Comparison of AUC was performed using the method proposed by DeLong et al.^[18]^ The Fisher exact test was used to compare independent groups for categorical variables. Nonparametric tests for trend were used for analysis across ordered groups. The McNemar test was used for paired comparison of proportions (sensitivity and specificity). A *P* < 0.05 was considered to indicate a statistically significant difference.

## 3 Results

### 3.1 Baseline characteristics

Of the 198 lesions, 125 (63.1%) were malignant, and 73 (36.9%) were benign (Table 1). The average of maximal diameter at conventional B-mode US was 19.50 ± 7.14 mm (range, 7–38 mm), without significant difference between malignant and benign lesions (20.89 ± 7.15 vs 17.07 ± 6.49 mm, *P* = 0.372).

**Table 1:**
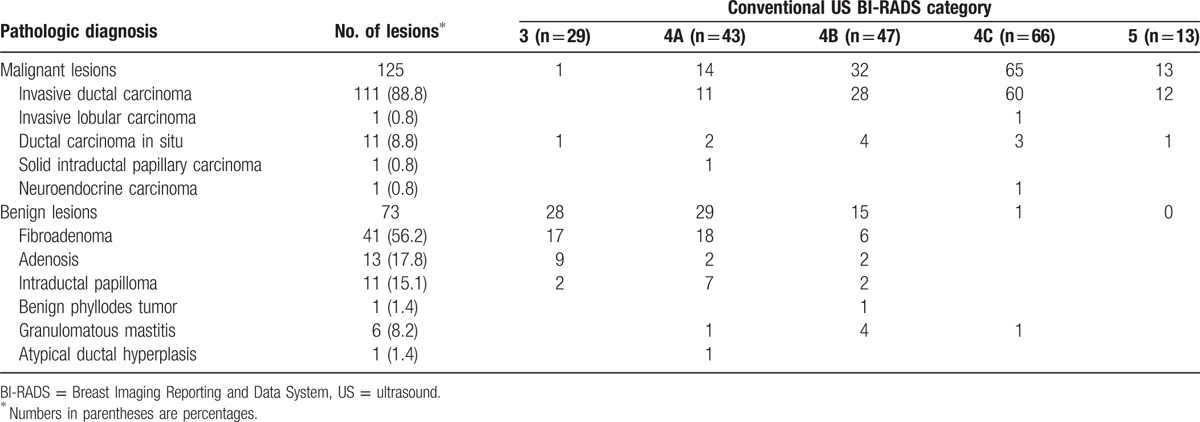
Pathologic diagnosis of 198 breast lesions and performance of conventional US.

### 3.2 Diagnostic performance of conventional B-mode US

The conventional US BI-RADS Category was shown in Table 1, and malignant rates were as follows: 3.4% (1/29) for BI-RADS Category 3, 32.6% (14/43) for BI-RADS Category 4A, 68.1% (32/47) for BI-RADS Category 4B, 98.5% (65/66) for BI-RADS Category 4C, and 100% (13/13) for BI-RADS Category 5. Overall sensitivity and specificity of conventional B-mode US were 88.0% (95% confidence interval (CI): 81.0–93.1) and 78.1% (95% CI: 66.9–86.9), respectively. The AUC was 0.913 (95% CI: 0.865–0.949), with the optimal cutoff value between Categories 4A and 4B.

### 3.3 Distribution of SWE color patterns of breast lesions in 3D SWE

**Figure 2:**
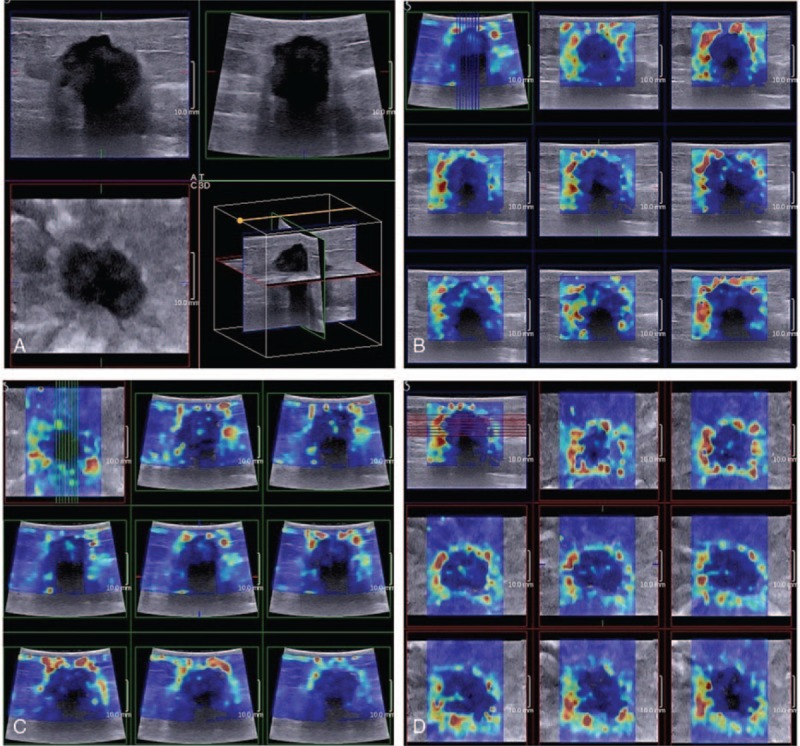
Images showed 3D B-mode and 3D shear wave elastography (SWE) of Grade II invasive ductal carcinoma of a 58-year-old woman. Image (A) showed 3D B-mode in multiplane mode (View A: transverse plane; View T: sagittal plane; and View C: coronal plane), with posterior acoustic attenuation in Views A and T while absent in View C. Image b to d showed 3D SWE of Views A, T, and C, respectively, with “Horseshoe” Pattern in Views A and T, which lacked of SWE information in part of the lesion and “Stiff Rim” Pattern in coronal plane (renamed as “Crater Sign” in our study) providing SWE information of the whole lesion.

The distribution of SWE color patterns of breast lesions, as divided into benign and malignant groups, was shown in Table 2. SWE color patterns of “No findings” and “Vertical Strips” constituted most part of benign lesions in Views A, T, and C (83.6%, 61/73; 82.2%, 60/73; and 78.1%, 57/73, respectively), while vast majority of malignant lesions showed the patterns of “Stiff Rim” and “Colored Lesions” (89.6%, 112/125; 90.4%, 113/125; and 97.6%, 122/125, respectively, for Views A, T, and C). A part of malignant lesions in View A (7.2%, 9/125) and View T (6.4%, 8/125) showed as “Horseshoe” Pattern, which all presented as Pattern of “Stiff Rim” in View C, named as “Crater Sign” in our study (Fig. 2) The distribution of SWE color patterns of malignant lesions was significantly different from that of benign lesions (*P* = 0.001).

**Table 2:**
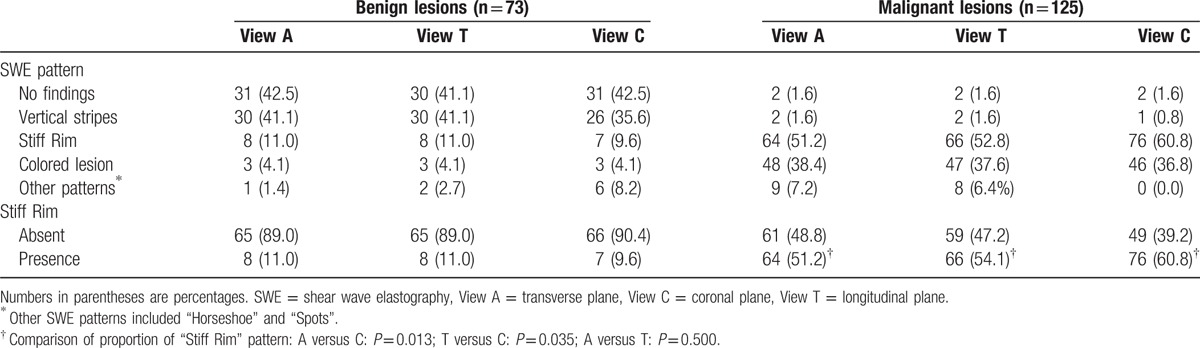
Distribution of color patterns of 3D SWE.

In malignant groups, “Stiff Rim” Pattern was significantly more frequently shown in View C (60.8%, 76/125) than in View A (51.2%, 64/125) (*P* = 0.013) and View T (54.1%, 66/125) (*P* = 0.035). In Views A, T, and C, the proportion of lesions showing “Stiff Rim” was significantly higher in IDC than in DCIS (55.0%, 61/111 vs 22.2%, 2/9, *P* = 0.020; 56.8%, 63/111 vs 22.2%, 2/9, *P* = 0.015; and 64.9%, 72/111 vs 22.2%, 2/9, *P* = 0.003, respectively, for Views A, T, and C), while in IDC lesions alone, this proportion was significantly higher in View C than in View A (*P* = 0.003) and View T (*P* = 0.012).

### 3.4 Diagnostic performance of SWE color patterns in 3D SWE

As compared with conventional BI-RADS US Category, the AUC for SWE color patterns in Views A, T, and C did not significantly improve (0.918, 95% CI: 0.869–0.953, *P* = 0.771; 0.916, 95% CI: 0.867–0.952, *P* = 0.758; and 0.918, 95% CI: 0.869–0.952, *P* = 0.924, respectively), while sensitivities of SWE color patterns significantly increased in Views A, T, and C (96.6% vs 88.0%, *P* = 0.035; 96.6% vs 88.0%, *P* = 0.035; and 97.6% vs 88.0%, *P* = 0.004, respectively), without significant difference in between (Table 3).

**Table 3:**
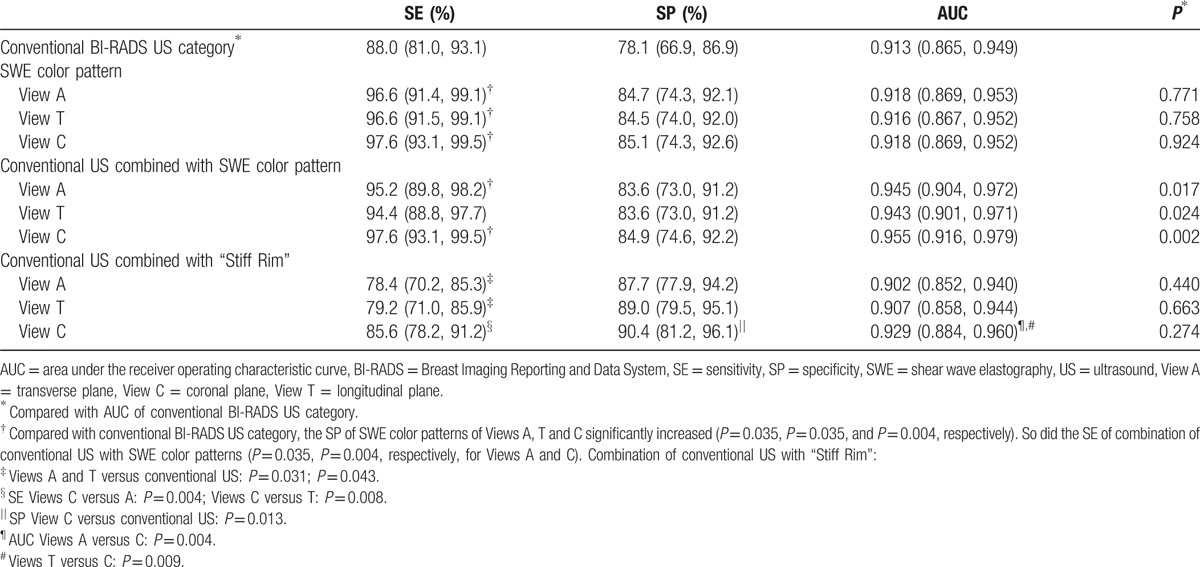
Diagnostic performance of conventional BI-RADS US category combined with 3D SWE color patterns and “Stiff Rim”.

### 3.5 Hypothetical effect of 3D SWE color patterns on diagnostic performance of BI-RADS assessment

According to the statistics above, the optimal cutoff of BI-RADS Category in differentiating benign from malignant was between Categories 4A and 4B. To assess the effect of 3D SWE color patterns on diagnostic performance of conventional US, lesions of conventional US BI-RADS Categories 4A and 4B were adjusted according to the SWE color patterns of Views A, T, and C. BI-RADS Category 4A was upgraded to 4B if SWE color pattern was “Stiff Rim” or “Colored Lesions”, while BI-RADS Category 4B was downgraded to 4A if SWE color pattern was “No findings” and “Vertical Stripes”. BI-RADS Category did not change with other patterns. The AUC for combination of conventional US with 3D SWE color patterns was significantly higher than that of conventional BI-RADS Category in Views A, T, and C (0.945, 95% CI: 0.904–0.972, *P* = 0.017; 0.943, 95% CI: 0.901–0.971, *P* = 0.024; and 0.955, 95% CI: 0.916–0.979, *P* = 0.002, respectively), without significant difference in between. As compared with conventional US, sensitivities of combination of conventional US with 3D SWE color patterns in Views A and C significantly increased (95.2%, *P* = 0.035 and 97.6%, *P* = 0.004), while that of View T did not show significant difference (94.4%, *P* = 0.057); specificities increased without statistical significance (*P* = 0.267, 0.267, and 0.146, respectively, for Views A, T, and C) (Table 3).

### 3.6 Hypothetical effect of “Stiff Rim” in 3D SWE on diagnostic performance of BI-RADS assessment

By adding “Stiff Rim” to lesions with conventional US BI-RADS Categories as 4A and 4B, Category 4A was upgraded to 4B if “Stiff Rim” was shown, otherwise Category 4B to 4A. AUC for combination of conventional US with “Stiff Rim” did not significantly change in any of 3 orthogonal planes comparing with conventional US, while significantly higher in View C (“Crater Sign”) (0.929, 95% CI: 0.884–0.960) than View A (0.902, 95% CI: 0.852–0.940) (*P* = 0.004) and View T (0.907, 95% CI: 0.858–0.944) (*P* = 0.009). Comparing with conventional US, specificity for combination of conventional US with “Stiff Rim” in View C (“Crater Sign”) significantly increased (90.4% vs 78.1%, *P* = 0.013), without significant change in sensitivity (85.6% vs 88.0%, *P* = 0.664), which was higher than sensitivities of View A (85.6% vs 78.4%, *P* = 0.004) and View T (85.6% vs 79.2%, *P* = 0.008) (Table 4).

**Table 4:**
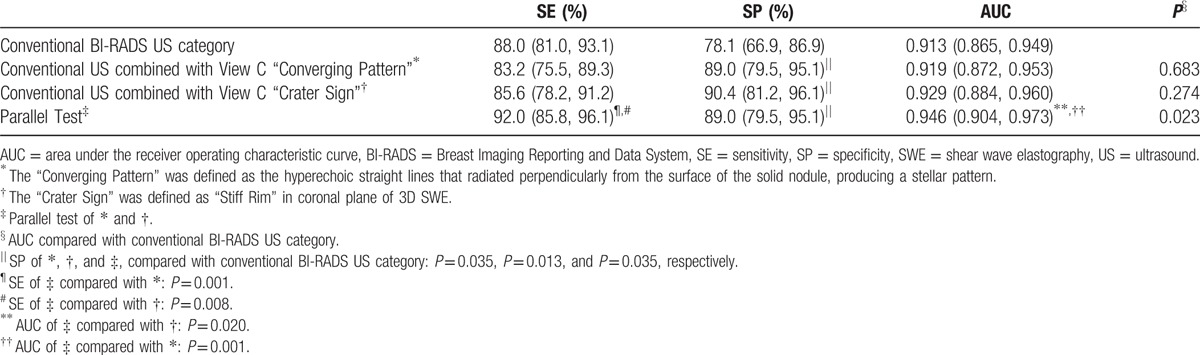
Diagnostic performance of “Converging Pattern”, “Crater Sign”, and parallel test.

### 3.7 Diagnostic performance of coronal plane, combining SWE with B-mode

In reconstructed coronal plane of 3D B-mode US, 73.6% (92/125) of malignant lesions presented as “Converging Pattern”, significantly higher than benign lesions (12.3%, 9/73, *P* = 0.001). Adjusting conventional BI-RADS US Categories 4A and 4B by “Converging Pattern”, specificity significantly increased (89.0% vs 78.1%, *P* = 0.035).

“Crater Sign”, defined as “Stiff Rim” in View C (coronal plane), was shown in 60.8% (76/125) of malignant lesions and significantly more frequent in IDC than DCIS (64.9%, 72/111 vs 22.2%, 2/9, *P* = 0.003). “Crater Sign” significantly increased specificity of diagnosing malignant and benign lesions (90.4% vs 89.0%, *P* = 0.013) without significant loss in sensitivity (85.6% vs 88.0%, *P* = 0.664).

The parallel test of “Converging Pattern” and “Crater Sign” adjusted BI-RADS Category significantly increased AUC (0.946, 95% CI: 0.904–0.973) comparing with conventional BI-RADS Category (0.913, 95% CI: 0.865–0.949) (*P* = 0.023), and both higher than “Converging Pattern” adjusted Category (0.919, 95% CI: 0.872–0.953, *P* = 0.001) and “Crater Sign” adjusted Category (0.919, 95% CI: 0.872–0.953, *P* = 0.001), yielding significantly higher sensitivity than the latter 2 methods alone (92.0% vs 83.2%, *P* = 0.001; 92.0% vs 85.6%, *P* = 0.008). The specificity did not show significant difference with that of either single method alone, but significantly increased compared with that of conventional US (89.0% vs 78.1%, *P* = 0.035) (Table 4).

## 4 Discussion

SWE is a brand new method of US elastography providing elastic information in real time, not relying on stress applied. The combination of 3D US and SWE was first realized by Supersonic Shear Imaging technique to provide volume elasticity information including reconstructed coronal plane. So far as we know, both the 2 published studies about 3D SWE focused on quantitative parameters of SWE, which both drew similar conclusions that diagnostic accuracy of 3D SWE was no better than 2D SWE,^[12,13]^ while in our study of 3D SWE, the important qualitative SWE feature—color pattern—was first involved.

Diagnostic performance of SWE color patterns have been demonstrated useful in breast lesions.^[5,6,8]^ In our study, SWE color patterns of malignant lesions were significantly different from benign lesions in 3D SWE, similar to previous studies of 2D SWE. Most malignant lesions tended to showed as “Stiff Rim” and “Colored Lesions” in transverse, sagittal, and coronal planes, and “Stiff Rim” was more frequently shown in coronal plane (60.8%) than in transverse (51.2%) and sagittal planes (54.1%) (*P* < 0.05), which aroused our notice of the probably higher value of coronal plane. The presence of “Stiff Rim” indicated high stiffness of the surrounding tissue in malignant lesions, which might be caused by the desmoplastic reaction or the infiltration of cancer cells into the peritumoral tissue.^[19,20]^ As indicated by Zhou et al,^[15]^ the “Stiff Rim” sign at less than 180 kPa significantly increased sensitivity without significant loss of specificity while providing similar AUC compared with conventional US. Similarly in our study, the sensitivity of 3D SWE color patterns in each view of the 3 orthogonal planes significantly increased, with similar specificity and AUC comparing with conventional US. What is new and worth mentioning is that BI-RADS Category adjusted by “Stiff Rim” in coronal plane yielded the highest AUC (0.929) and sensitivity (85.6%) among the 3 orthogonal planes (*P* < 0.05) and significantly higher specificity (90.4%) than conventional US (78.1%). This result probably indicated higher value of “Stiff Rim” in coronal plane, which was renamed as “Crater Sign” in our study since malignant breast lesions more frequently showed as “Converging Pattern” in coronal plane of 3D B-mode images.

In practice, architectural distortion and spiculation of breast lesions are important features that highly indicate malignancy, which could be clearly observed in coronal plane of 3D B-mode images and characterized by a similar “Converging Pattern”.^[21–23]^ As commonly recognized as a manifestation of aggressive cancer,^[24]^ “Converging Pattern” might be caused by traction of Cooper ligaments into a neoplasm and desmoplastic reaction induced by the invasion of cancer cells and thus presented as hyperechoic bands of fibrous tissue converging toward the hypoechoic mass. In our study, a majority of malignant lesions presented as “Converging Pattern” in coronal planes, consistent with previous studies. Combination of conventional US with “Converging Pattern” significantly increased specificity (*P* < 0.05). Based on “Converging Pattern”, the colored “Stiff Rim” surrounding the lesions in coronal plane looked like the crater and thus was renamed as “Crater Sign” in our study, which was first proposed so far. Parallel test of “Converging Pattern” and “Crater Sign” yielded significantly higher sensitivity than either of the 2, and significantly higher specificity than conventional US.

Better performance of “Stiff Rim” in coronal plane (Crater Sign) than transverse and sagittal planes may be attributed to the significantly higher frequency of “Stiff Rim” Pattern, part of which presented as “Horseshoe” in transverse and sagittal planes, without the elasticity information of part of the tumor edge (usually the deep side), which was probably due to significant acoustic attenuation in the posterior part of the lesion on B-mode images. Since SWE features was determined according to the slice showing maximal diameter, the reconstructed coronal plane is consequently less affected by acoustic attenuation and seems to provide more information about SWE of the whole lesion, including SWE color pattern mentioned above and even other qualitative features such as shape of lesion, heterogeneity of elasticity, and quantitative parameters such as maximum, minimum, and mean value of elasticity. The discovery of “Crater Sign” might draw attention to the diagnostic value of SWE information of coronal plane, which might be lost in transverse and sagittal planes in some lesions.

In malignant groups, there were a part of lesions presented as “Colored Lesions”, coronal plane did not show greater advantages over transverse and sagittal planes among this group of lesions. However, the pattern of “Stiff Rim” constituted of the majority of malignant group, so diagnostic performance of SWE color pattern of “Crater Sign” in coronal plane was worth highlighting.

This study also had some limitations. First, selection bias may exist because of retrospective study, and patients enrolled were scheduled for surgical excision, which could explain the higher malignant rate of BI-RADS 4A Categories of conventional US than that of ACR BIRADS-US. By reviewing those malignant lesions assessed as BI-RADS 4A Categories by conventional US, we found that most lesions lacked of malignant features, such as irregular shapes, blurred edges, posterior acoustic attenuation, calcifications, and hypervascularity. However, after combining with 3D SWE, quite a part of these lesions showed as “Stiff Rim” or “Colored Lesion” and BI-RADS Categories were upregulated. Second, large masses which could not be covered by the maximal range of SWE color overlay were excluded in our study, while there were controversy about relationship between the size of lesions and the presence of “Coverging Pattern” in the coronal plane.^[25,26]^ Jiang et al^[25]^ found that breast tumors with “Coverging Pattern” were more likely to be small, while Lamb et al^[26]^ demonstrated no significant correlation between the two. If correlation exists, the exclusion of lesions of large size may cause selection bias to study cohort, but it would not change the result of higher frequency of “Stiff Rim” in coronal plane than transverse and sagittal planes of 3D SWE. Third, 3D technique probably produces more artifacts owing to the compression factor of heavier 3D transducer and movement during image acquisition.^[27]^ So the radiologists were instructed to very lightly apply the transducer to avoid compression and kept still during 3D B-mode and SWE data acquisition, and generous amount of coupling agent was applied. When assessing color pattern of 3D SWE, the slice showing the maximal diameter was selected in all the 3 orthogonal planes, thus we consider that color pattern of coronal plane, from which the slice selected was not so near to skin, may be less influenced by artifacts than transverse and sagittal planes.

In conclusion, combination of conventional US with 3D SWE color patterns significantly increased diagnostic accuracy of differentiating benign from malignant lesions in all the 3 orthogonal planes. Sensitivities and AUC of combining conventional US with 3D SWE color patterns significantly increased. Owing to significantly higher frequency of “Stiff Rim” in coronal plane, which was renamed as “Crater Sign” in this study, coronal plane yielded the highest AUC and sensitivity of diagnosing malignant lesions among the 3 orthogonal planes and significantly higher specificity than conventional US. Further perspective studies of large sample would be needed for validation of our results.
